# Supernovae Detection with Fully Convolutional One-Stage Framework

**DOI:** 10.3390/s21051926

**Published:** 2021-03-09

**Authors:** Kai Yin, Juncheng Jia, Xing Gao, Tianrui Sun, Zhengyin Zhou

**Affiliations:** 1 School of Computer Science and Technology, Soochow University, Suzhou 215006, China; 20195427003@stu.suda.edu.cn (K.Y.); 20205227004@stu.suda.edu.cn (Z.Z.); 2 Collaborative Innovation Center of Novel Software Technology and Industrialization, Nanjing 210000, China; 3 Xinjiang Astronomical Observatory, Chinese Academy of Sciences, Urumqi 830011, China; gaoxing@nao.cas.cn; 4 Purple Mountain Observatory, Chinese Academy of Sciences, Nanjing 210023, China; trsun@pmo.ac.cn; 5 School of Astronomy and Space Science, University of Science and Technology of China, Hefei 230026, China

**Keywords:** image processing, data analysis, sky surveys, supernova, object detection

## Abstract

A series of sky surveys were launched in search of supernovae and generated a tremendous amount of data, which pushed astronomy into a new era of big data. However, it can be a disastrous burden to manually identify and report supernovae, because such data have huge quantity and sparse positives. While the traditional machine learning methods can be used to deal with such data, deep learning methods such as Convolutional Neural Networks demonstrate more powerful adaptability in this area. However, most data in the existing works are either simulated or without generality. How do the state-of-the-art object detection algorithms work on real supernova data is largely unknown, which greatly hinders the development of this field. Furthermore, the existing works of supernovae classification usually assume the input images are properly cropped with a single candidate located in the center, which is not true for our dataset. Besides, the performance of existing detection algorithms can still be improved for the supernovae detection task. To address these problems, we collected and organized all the known objectives of the Panoramic Survey Telescope and Rapid Response System (Pan-STARRS) and the Popular Supernova Project (PSP), resulting in two datasets, and then compared several detection algorithms on them. After that, the selected Fully Convolutional One-Stage (FCOS) method is used as the baseline and further improved with data augmentation, attention mechanism, and small object detection technique. Extensive experiments demonstrate the great performance enhancement of our detection algorithm with the new datasets.

## 1. Introduction

A series of sky surveys, such as the Dark Energy Surveys [1], the Panoramic Survey Telescope and Rapid Response System (Pan-STARRS) [2], the Large Synoptic Survey Telescope (LSST) [3] and so on, were launched in search of transient astronomical events that are astronomical sources or phenomena with limited duration such as supernovae. These sky surveys generated a tremendous amount of data, which pushed astronomy into a new era of big data. It is important to detect supernovae quickly and accurately. However, many data processing pipelines for these sky surveys rely strongly on human inspection. It can be a disastrous burden to manually identify and report supernovae, because such data have huge quantity and sparse positives. So there is an urgent need for a fast and automatic supernovae detection system.

There are some existing works leveraging machine learning methods in this area. Some researches dedicating to supernovae classification, that is, to differentiate supernovae or artifacts, have already reached relatively high performance. Du Buisson et al. [4] tested and compared many traditional machine learning methods for supernovae classification. The Random Forest (RF) [4,5] algorithm performs best among traditional machine learning methods. With the development of deep learning methods such as Convolutional Neural Network (CNN), they show strong adaptability to deal with massive astronomical data. Cabrera-Vives et al. [6] proposed a CNN classifier that outperforms the Random Forest model. Another direction of this study is to analyze the variation of supernova spectral sequences. Again, deep learning methods represented by Recurrent Neural Networks (RNN) have achieved very good results in this task. Muthukrishna et al. [7] introduced the Real-time Automated Photometric Identification (RAPID) architecture and implemented fine-grade supernovae classification. Burke et al. [8] applied object detection to the astronomical source classification.

However, there are still some limitations of the existing works. A review article in Nature [9] argued that researchers are facing a challenge in the supernovae classification: Dataset is in the absence of statistically representative of the supernova. In fact, most data in the existing works either are simulated or lack generality. Furthermore, most of the existing works study supernovae classification, which assumes the input images are properly cropped with a single candidate located in the center and returns the label or class of the candidate. However, for images in some datasets, the candidates may not be centrally located, or there are multiple candidates in a single image. These cases can be regarded as supernovae detection tasks, which should return locations of detected supernovae. Besides, the performance of existing detection algorithms for general object detection tasks can still be improved for the supernovae detection task.

To address the above two issues, in our work we constructed two new datasets for supernovae detection, which consist of data from the Pan-STARRS and the Popular Supernova Project (PSP). The typical detection method is to compare the difference between the two images taken in the same azimuth but at different time. So all the image data we collected are difference images. The data from the Pan-STARRS have relatively high quality, while the data in the PSP are defective images from the public observations. Most of the PSP images have a relatively low signal-to-noise ratio (SNR), due to factors like bad weather, moonlight, equatorial mount failure, and so on. To detect supernovae in our datasets, we first compared several mainstream object detection algorithms and chose the best one, that is, the Fully Convolutional One-Stage (FCOS) algorithm [10], and then improved FCOS on this specific task.

The main goal of this paper is to develop an automatic supernovae detection framework with object detection techniques and thus accelerate the processing of observation data. Our contributions in this paper are summarised as follows:

- We made a labeled dataset, which consists of 12,447 images from the Pan-STARRS with all supernovae labeled.
- We made a labeled dataset, which consists of 716 images from the PSP with all supernovae labeled. Considering the amount of samples in the PSP dataset is too small, we also used the data augmentation technique on this dataset.
- We compared several detection algorithms on both datasets. The FCOS method with better performance is used as the baseline method. In addition, the FCOS algorithm is improved with different techniques, such as data augmentation, attention mechanism, and increasing input size. To verify the challenge mentioned in the [9], we have tested the performance of model training for the hybrid datasets with different blending ratios.

The rest of this paper is structured as follows—the relevant existing works of supernovae classification, object detection, and attention mechanism are reviewed in Section 2. Then we introduce our datasets in Section 3. In Section 4, the supernovae detection framework and improvements are presented. In Section 5, we conduct extensive experiments to evaluate the detection methods on the two datasets. Some other methods are discussed in Section 6. Finally, we summarize our work in Section 7.

## 2. Related Works

### 2.1. Supernovae Classification

In recent years, Machine Learning (ML) based algorithms have just been applied in supernovae classification, which is to differentiate types of supernovae. Du Buisson et al. [4] widely studied the performance of different ML algorithms on the 2nd and 3rd years of the Sloan Digital Sky Survey (SDSS)-II supernova survey. They extracted the features with Principal Component Analysis (PCA) and the normalized data are cropped by 31×31 pixels at the center. They tested and compared many algorithms on the processed data. The result shows that the Random Forest algorithm has the best performance in these classifiers. The Random Forest (RF) is an ensemble classifier. In their work, the optimal splitting points are selected from a random sample of features at every node, and then they split the data into different decision trees. Moreover, they studied the Cohen’s kappa coefficient [11] between these algorithms and it turns out that these algorithms can at most reach moderate agreements.

Cabrera-Vives et al. [12] proposed a Convolutional Neural Network (CNN) classifier to classify different objects in images as transients or artifacts. They designed a neural network with 2 convolutional layers and 2 pooling layers, which are followed by 2 fully connected layers to resize the output into a 1×2 vector. Each element of the vector represents the possibility of transients or artifacts. They achieved higher performance than the RF model. Also based on CNN, Kimura et al. [13] proposed a deeper neural network with 3 convolutional layers combined with 5 channels for different frequency bands on a self-built dataset.

In 2017, Cabrera-Vives et al. [6] proposed a rotation-invariant CNN model which outperforms their former work. Their neural network has four branches. The candidates will be rotated in 90°, 180°, 270° first and then four images will be fed into each branch. Each branch includes five convolutional layers and two pooling layers. The model concatenates the output of four branches and uses fully connected layers to produce a 1×2 vector output representing the possibilities of transients and artifacts. In the next year, Reyes et al. [14] enhanced this model by proposing a measure that assesses the rotational invariant effect on the Layerwise Relevance Propagation (LRP) relevance heatmaps.

The Recurrent Neural Networks (RNNs) [15] classify supernovae from a different perspective. RNNs can process complicated sequential data and are capable of learning about the light curves of supernovae. Charnock et al. [16] tested the performance of the Long Short-Term Memory (LSTM) [15] and the Gated Recurrent Unit (GRU) [17,18]. They have achieved better results than other algorithms. Muthukrishna et al. [7] introduced the RAPID architecture using GRUs to achieve fine-grade supernovae classification. Moller et al. [19] proposed to use RNN with the Bayesian training method. They compared their work with Support Vector Machine (SVM) [20], CNN, and non-Bayesian RNN.

All image classification based methods mentioned above encountered a problem—the candidates have to be selected and cropped in the center approximately. If the candidate is located off-center in the image, the performance of the model will decrease. Burke et al. [8] applied object detection on the astronomical source classification. Their object detection framework is based on the Mask-RCNN [21] and treat the classification task as a star/galaxy detection task and achieved performance with 92% precision and 80% recall on stars and 98% precision and 80% recall on galaxies. Because of the object detection, their model can predict the category and location of the target when images with different input sizes are input.

### 2.2. Object Detection

Unlike common neural networks, the object detectors have an extra head predicting class and bounding box positions in addition to the backbones. The detection frameworks are classified into one-stage detectors and two-stage detectors. One-stage detectors output the confidence of the classes and the positions directly, while two-stage detectors have to propose the possible position of the objects and then classify them. Usually, the two-stage detectors have higher accuracy but are slower than the one-stage detectors.

The representative two-stage object detection networks are R-CNN [22] based detectors. The Faster-RCNN [23] is one of the most excellent detectors for a long time. The Faster-RCNN uses an extra layer called Region Proposal Network (RPN) to provide objects for the classification network. The RPN layer provides 2000 anchors and chooses the most possible targets for classification, and is much more efficient than the Selective Search algorithm. At the end of the RPN layer, the author applies an ROI pooling layer to map the proposed targets to the feature map. Because most of the works are finished by neural networks, the detection speed and accuracy have been significantly improved. The Mask-RCNN [21], the segmentation version of the Faster-RCNN, is also used in Burke’s work [8] and has great performance.

On the other side, the one-stage detectors such as You Only Look Once (YOLO) [24], RetinaNet [25], CornerNet [26], FCOS [10] and so on, have caught up with two-stage detectors recently. The main idea of YOLO algorithms is to assign the classification task to the pre-defined grids. The images are divided into S×S grids and the bounding box is determined by the grid with the highest IOU. The detecting speed of the YOLOv1 can reach 45 fps. In these YOLO architectures, YOLOv3 [27] is the most widely used detectors on a variety of devices for its speed and accuracy. The author increases the number of the backbone layer to 53 (Darknet53 [27]) and uses an independent logistic regression classifier instead of softmax. The YOLOv3 runs even faster and keeps similar performance to the SSD [28]. The YOLOv3 has a relatively better performance than YOLOv1 for detecting small objects. The FCOS [10] attempts to achieve high accuracy without pre-defined anchors, which eliminates several limitations caused by anchors. It uses the Center-ness layer to suppress the generation of these negative bounding boxes, which greatly improves the recall.

### 2.3. Attention Mechanism

The attention mechanism has been proposed for a long time, but the seminal work [29,30] makes this technology widely known. The attention mechanism is widely used in recurrent and convolutional neural networks. The main idea of this technique is to redistribute the weights so that the neural network can focus more on specific features. There are two types of attention mechanism, that is, hard attention and soft attention. Hard attention directly sets the weight of the region of no interest to 0, while soft attention determines the weight distribution in the range between 0 and 1 smoothly and increases the weight of the focused area. Soft attention is more suitable for gradient calculating and therefore is widely used in neural network research. In 2017, Hu et al. [31] proposed an attention module called Squeeze-and-Excitation (SE) block, which enables neural networks to focus on different channels. The SE module calculates representative values for different channels and multiplies these values to the channels to redistribute weights. Their later work, the SENet, won the championship of the ImageNet. Li et al. [32] presented an attention structure that can focus on the channels generated with different kernel sizes, or feature maps with different receptive fields in other words. In addition to the channel-wise attention mechanism, Wang et al. [33] tried to deal with the information missing problem and proposed a spatial attention module. Their module is a parameter-free spatial attention layer generating positional weight and activates back to the model. Woo et al. combined the channel-based attention and the spatial attention and proposed the Convolutional Block Attention Module (CBAM) [34]. The CBAM achieves better results on the ImageNet-1k dataset. In [35], Chen et al. presented a method that integrates RetinaNet and attention mechanism to generate the video synopsis for long captured videos. In their experiments, the combined attention method improved the Average Precision (AP) of detection by about 3%. The Center-Mask [36] is an efficient anchor-free instance segmentation algorithm based on the FCOS, which uses an attention-guided mask (SAG-Mask) in the detectors. They used effective Squeeze-and-Excitation (eSE) instead of the original SE module and achieved the highest mask AP of 38.3%.

## 3. Datasets

Most of the data used by the existing works, as mentioned in Section 2, are simulated data or partially generated by simulation. In [7,8,12,16,19], the positive candidates for training are simulated. In [13,14], the authors simulated observation fluctuations such as flux and SNR variation. Currently, there is a lack of publicly available real data for the supernovae detection research, which hinders the development of this field. In this section, we built two datasets—the PS1-SN dataset and the PSP dataset. All the images of both datasets come from real observation instead of generating by simulation. Different from the simulated data, the images in our datasets include various defects caused by the actual observation process.

### 3.1. PS1-SN Dataset

Our first dataset is referred to as the Pan-STARRS1 SuperNova (PS1-SN) dataset comes from the pipeline of the Panoramic Survey Telescope and Rapid Response System for Supernovae (https://star.pst.qub.ac.uk/ps1threepi/psdb/, accessed on 26 May 2020), a system for wide-field astronomical imaging.

**Figure 1:**
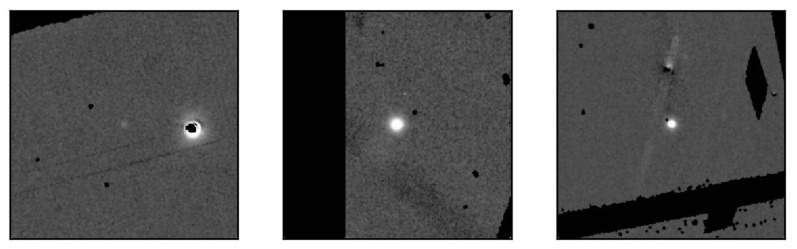
Sample images in the Pan-STARRS1 SuperNova (PS1-SN) dataset.

We collect about 12,447 difference images from the known object list. We have labeled all the supernovae in these images and constructed the PS1-SN dataset. Some samples in this dataset are displayed in Figure 1. All of the images have the same size of 302×302 and most of the targets are in the center of the images. The images from this program have relatively high quality. They are evenly exposed and their backgrounds are free of debris. As a result, supernovae are very obvious in this dataset. Compared with the PSP-SN dataset to be introduced later, detection models trained with the PS1-SN dataset result in better performance.

### 3.2. PSP-SN Dataset

**Figure 2:**
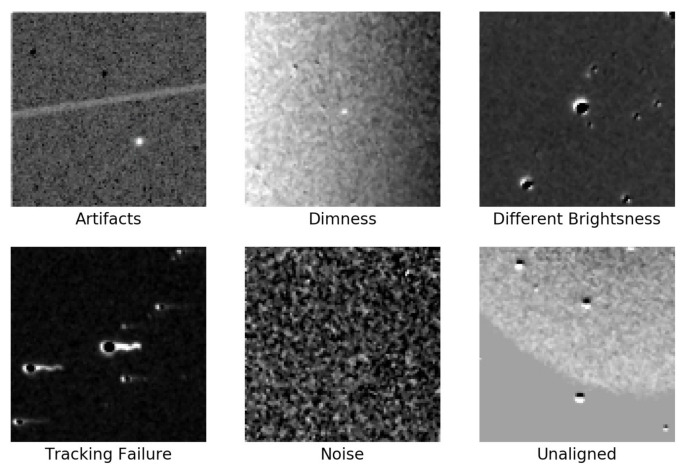
Defections in the PSP-SN dataset.

The PSP-SN dataset consists of 716 images. These images come from the Popular Supernova Project (PSP) hold by the Xingming Observatory, a sky survey program open to the public (http://psp.china-vo.org/, accessed on 14 November 2019). The targets in the PSP-SN dataset are all publicly reported and officially confirmed supernovae. Most of the images in this dataset have one single confirmed target. All images are cropped into different sizes according to the azimuth. The PSP-SN dataset has the following characteristics:

- Most of the images have defects. Typical defects in this dataset are shown in Figure 2, which are caused by low SNR rate, artifacts, equatorial mounts failure, unaligned, different brightness, bad weather, and so on. While these defects increase the variety of data, they also bring about complexity to achieve high detection accuracy.
- The number of samples provided by the PSP project is insufficient. Deep learning methods usually require a large number of samples to train models with high accuracy.
- The images from the PSP are cropped into different sizes. The image sizes of the PSP range from 288×288 to 950×950.

**Figure 3:**
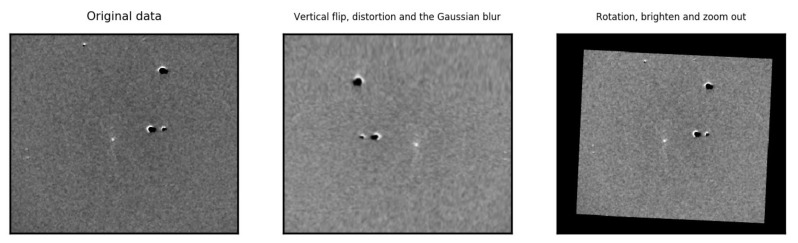
Sample images in the Popular Supernova Project augmented (PSP-Aug)dataset.

In the later experiments, we will apply data augmentation to the PSP-SN dataset to improve the performance of the detection model. We augmented the original PSP-SN dataset offline with Gaussian blur, rotation, distortion, horizontal, vertical flip and so on. Some augmented samples are displayed in Figure 3. Eventually, we obtain an augmented version of the PSP-SN dataset with 12,384 images. For convenience, the original PSP-SN and the augmented PSP-SN dataset will be referred to as the PSP-Ori and the PSP-Aug later.

## 4. Methods

**Figure 4:**
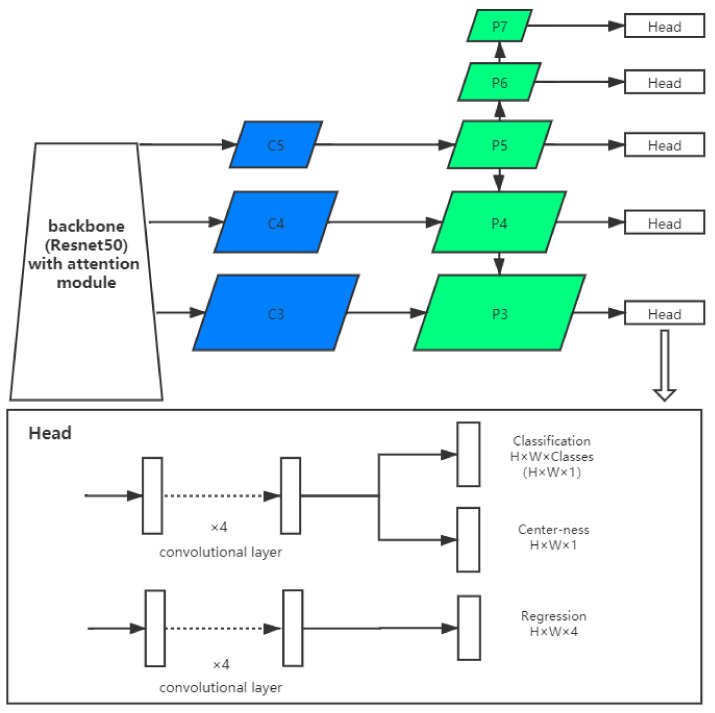
The structure of the Fully Convolutional One-Stage (FCOS) with attention blocks.

Our supernovae detection neural network is based on the FCOS framework enhanced by attention mechanism, which is displayed in Figure 4. In this section, we explain the basic FCOS framework, the enhance with attention mechanism and manipulation with image input size.

### 4.1. The FCOS Framework

The FCOS [10] is an anchor-free detector, since it predicts a 4-dimensional vector denoted as V(l,t,r,b), which contains the distances to left, top, right, bottom of each position. After the feature maps of different stages in the pyramid are extracted, they are sent to the FCOS head for further processing. The FCOS head consists of the regression branch and classification branch, both of which contain 4 convolutional layers. These convolutional layers transform the weights into classification vector and regression vector. To enhance the performance, the weights of the off-center bounding boxes are suppressed by a layer called the Center-ness. The Center-ness is a parallel branch to the classification output. The Center-ness layer uses the following formula to reduce the weight of the edge area and can greatly improve the recall of the FCOS.

(1) $$\mathrm{Center}-\mathrm{ness}=\sqrt{\frac{min(l^{*},r^{*})}{max(l^{*},r^{*})}\times \frac{min(t^{*},b^{*})}{max(t^{*},b^{*})}}\;,$$

where $l^{*},r^{*},t^{*},b^{*}$ denotes the predicted elements of V(l,t,r,b). The Center-ness produces a vector of the same size as the feature image. The value of the center in this vector is 1, and gradually reduces to 0 towards the edge. The vector and classification results are multiplied by the predicted elements to get the centralized classification result.

### 4.2. The Attention Module

**Figure 5:**
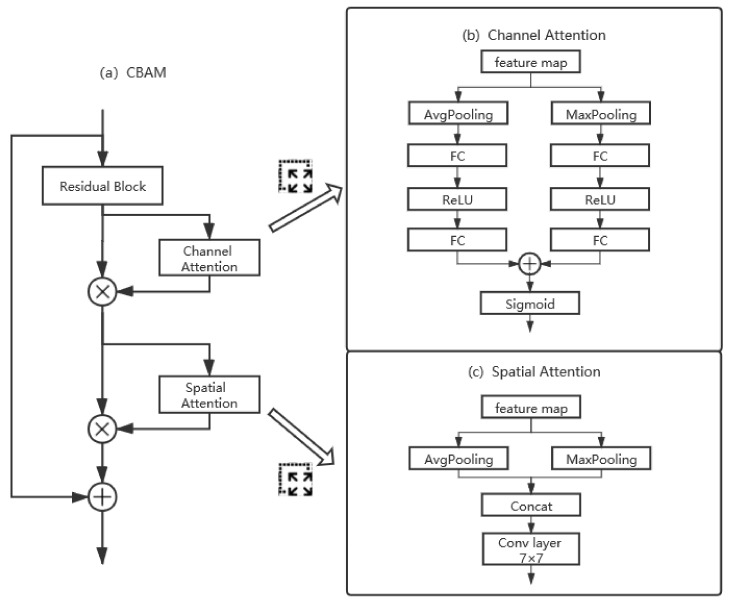
The structure of the attention blocks: (**a**) structure of CBAM, (**b**) structure of channel attention, (**c**) structure of spatial attention.

The attention mechanism is usually used to increase the weights of the useful features. There are 3 kinds of attention blocks tested in our paper: the channel-based attention (Squeeze and Excitation, SE) [31], spatial-based attention, and the combination of these two attention blocks (Convolutional Block Attention Module, CBAM) [34]. These modules are displayed in Figure 5.

The channel-based attention gives different weights to different channels. The channel-based attention formula is listed below:

(2) $$\mathrm{SE}\left(F_{in}\right)=\sigma \left(\mathrm{FC}\left(\delta \left(\mathrm{FC}\left(\mathrm{AvgPooling}\left(F_{in}\right)\right)\right)\right)\right).$$

Here, we have $F_{in}\in R^{H\times W\times C}$ as the input for the *i*th layer. First, a global average pooling (AvgPooling) operation is applied to generate a squeezed 1×C vector and each element of the squeezed vector represents a channel-wise value. Then, a fully connected (FC) layer is used to learn the nonlinear combination characteristics. The SE module uses the ReLU function denoted as δ to activate these representative channel-wise values and the range of output values is limited to [0, 1] with a sigmoid function denoted as σ. Squeezed and excited vector can inform the neural network which channels should be focused on. In this paper, we use channel attention (CA) with both average pooling and maximum pooling (MaxPooling) as follows:

(3) $$\mathrm{CA}\left(F_{in}\right)=\sigma (\mathrm{FC}\left(\delta \left(\mathrm{FC}\left(\mathrm{AvgPooling}\left(F_{in}\right)\right)\right)\right)+\mathrm{FC}\left(\delta \left(\mathrm{FC}\left(\mathrm{MaxPooling}\left(F_{in}\right)\right)\right)\right)).$$

Eventually, the *i*th layer feature map is multiplied with the vector to obtain the output weighted features:

(4) $$\begin{array}{c} F_{out}=\mathrm{CA}\left(F_{in}\right)\times F_{in}. \end{array}$$

The second attention we tested in our architecture is the spatial attention. The main idea of spatial attention is to generate a weighted feature map to represent the importance of different positions crossing channels. The summarized spatial attention is listed below:

(5) $$\begin{array}{c} \mathrm{SA}\left(F_{in}\right)={\mathrm{Conv}}_{7\times 7}\left(\mathrm{AvgPooling}\left(F_{in}\right)|\mathrm{MaxPooling}\left(F_{in}\right)\right), \end{array}$$

where notation “|” is the operation of concatenation, and Conv${}_{7\times 7}$ denotes a convolutional layer with a kernel size of 7×7. As in [34], an average pooling and maximum pooling are used to combine all the channels, then the output feature map is followed by a convolutional layer with a kernel size of 7×7 to calculate positional feature map. The final output is then:

(6) $$\begin{array}{c} F_{out}=\mathrm{SA}\left(F_{in}\right)\times F_{in}. \end{array}$$

The last attention is the combination of the two methods mentioned above, that is, CBAM. The CBAM uses the SE module first to generate channel attention, and then uses the spatial attention mechanism to generate positional attention. The shortcut links the input layer directly to the output layer:

(7) $$\begin{array}{c} F_{out}=\mathrm{CBAM}\left(F_{in}\right)=\mathrm{SA}\left(\mathrm{CA}\left(F_{in}\right)\times F_{in}\right)\times F_{in}. \end{array}$$

### 4.3. Increase Input Size

According to our analysis, most of the targets in our datasets are small objects (smaller than 32×32 pixels). Therefore, small object detection techniques are applied in our architecture. The size of the feature map becomes smaller because of the convolution operation and this phenomenon is more serious in deep convolution networks. The direct result of this phenomenon is that small targets lose most of the details at the end of deep convolutional neural networks. Therefore, the performance of the detector for small object detection is usually the bottleneck of the detection task. One of the basic techniques is to increase the sizes of input images. We initially set the image input size as 416×416, then increase to 800×800, and finally to 960×960.

## 5. Experiment

In this section, we first compare different object detection algorithms on the datasets and select the best one as the baseline. Then we improve the selected algorithm on the PSP dataset with some of the small object detection techniques.

### 5.1. Model Selection

We compare Deep-Hits (DH) [12], Cycling Average Pooling (CAP) [14], YOLOv3 and FCOS during model selection, as shown in Table 1. DH and CAP are designed specifically for supernovae detection, while YOLOv3 and FCOS are used for general object detection. First, we evaluate the DH model and the CAP model on the PS1-SN dataset. The original input size of the two rotation invariant models is 21 × 21, so we crop images in the PS1-SN dataset at the center. Then we generate negative samples by selecting the background, which produces about 12,000 negative samples. The test result shows that both of the models have high detection performance on the PS1-SN dataset. For the object detection model, we test YOLOv3 and FCOS on both the PS1-SN dataset and the PSP-Ori dataset. Both methods are tested with an original input size, which is much larger than 21 × 21. We fine-tune the training parameters for two models separately to achieve their best performance, as shown in Table 2. For the PS1-SN dataset, the two models perform similarly, where YOLOv3 has slightly higher precision and FCOS has a slightly higher recall. Both methods outperform the DH and CAP model. For the PSP-Ori dataset, all metrics are much lower. Since F1 scores of FCOS are higher than YOLOv3, we focus on the FCOS as our baseline.

### 5.2. Performance Enhancement

We use several improvement techniques to achieve a better performance on this task. According to the previous results, there is little improvement space for PS1-SN, where the performance on PSP-Ori is not good enough. Therefore, we use PSP datasets for evaluation in this part. As shown in Table 3, the data augmentation improves the performance a lot. To make the neural network focus on the feature of interest, we apply the attention mechanism to the FCOS. The training parameters are selected as follows—the batch size is all set to 8, the weight decay is $1\times 10^{-4}$, the learning rate is initialized to $1\times 10^{-3}$ and is reduced by a factor of 10 at the iteration of 14,000 and 20,000. We trained all methods in 25,000 iterations. We have tested three kinds of attention mechanisms, that is, the channel attention, the spatial attention, and the CBAM, with different input sizes. When the input size is 416×416, all attention mechanisms produce a negative effect on the performance. We then try to increase the size of the input image, which begins to work out. At first, the performance improvement is not obvious when the maximum image size is 800×800. When the maximum size is further increased to 960×960, the performance is enhanced. The spatial attention performs best this time, while the other methods still have the same or negative effect.

We also investigate the effect of the above enhancements on the YOLOv3, by conducting similar experiments of YOLOv3 as FCOS. As shown in Table 4, the detection performance of YOLOv3 improves with the size of the input image, but the improvement is not obvious when the input size changes from 800 to 960. The CBAM is counterproductive in the results, and the effects of spatial and channel attention are not obvious. Comparing Table 3 and Table 4, we can conclude that the enhanced FCOS achieves a better performance on the PSP-SN dataset.

**Figure 6:**
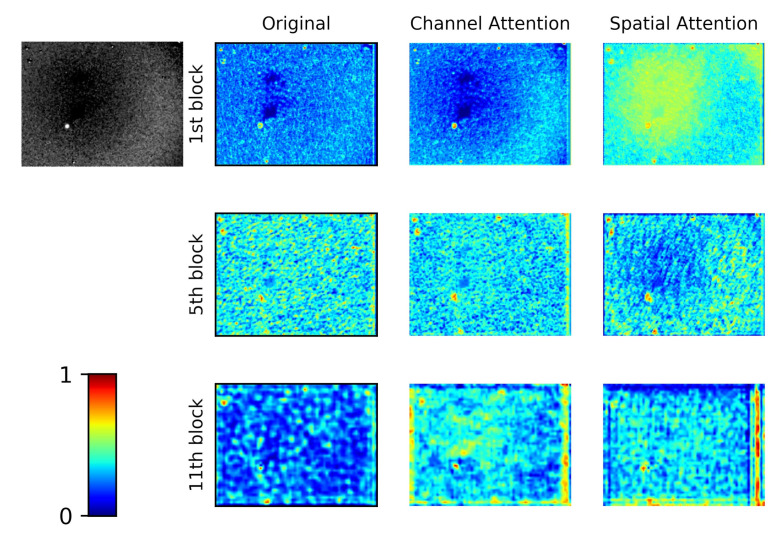
The feature map visualization with different attention blocks of FCOS.

The network visualization heat map displays the effects of channel attention and spatial attention. The data from the heat map comes from the output right after the attention module or residual blocks. We extract the feature map from the 1st, 5th, and 11th residual block of the enhanced FCOS. As Figure 6 shows, the attention module successfully attaches more weights to the target than the original network. Specifically, the advanced networks are more interested in regions of candidate supernovae.

## 6. Discussion

We list some methods that we tried but that did not work here. These methods include some neural network module designs and hybrid dataset techniques. Though these methods do not have a positive influence on the supernovae detection task, they still provide some ideas for solving relevant problems.

- Upsampling: As described in Section 3, supernovae detection suffers from feature loss in deep convolutional neural networks. We tried upsampling for the feature map with a ratio of 1.2 at the output of each feature pyramid stage and reduced the size shrinkage of the feature map to some extent. However, such a method did not improve much performance.
- Hybrid dataset: This idea is inspired by the article [9]. The author argues that training data for the relevant research lacks statistical representation. To figure out whether the practical representative samples can enhance the performance or not, we blend the two datasets of PSP-Aug and PS1-SN as a hybrid dataset. We test the performance of FCOS with two blending ratios of PSP-Aug to PS1-SN, which are 1:20 and 1:1. The models trained with the hybrid dataset are also fine-tuned to achieve the best performance and the results are listed in Table 5. When the blending ratio is increased, the performance with the PSP-Aug decreases severely while the performance with the PS1-SN does not change too much. This result shows that the detection architecture tends to have good performance on the data with low complexity, while causes performance degradation on the complex data.

**Figure 7:**
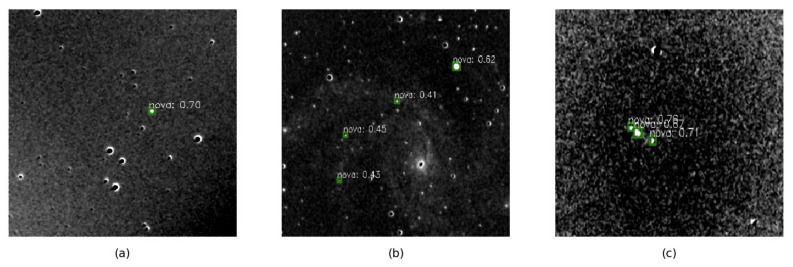
The unaligned images cause false positive predictions. Green box indicates the location of the supernova. The classification result and confidence is labeled above the green box. (**a**) The model doesn’t mistake unaligned stars as supernovae. The model can discover the features of unaligned stars to some extent. (**b**) Unaligned stars are mistaken for supernovae. (**c**) Unaligned edges of galaxies are mistaken for supernovae.

**Figure 8:**
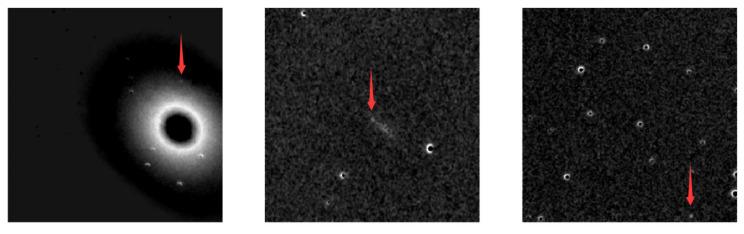
The dim targets cause false negative predictions. Red arrow points to the dim supernova in the image.

Another problem to be discussed is the ability of the model to detect different types of defect images. We save the detection results of the testing set for analysis. Figure 7 shows several examples of false positive results. The green bounding-boxes mark the targets, and the category and confidence are labeled beside the bounding-boxes. Although the model can discover the features of unaligned stars to some extent, as shown in Figure 7a, the model produces most of the false positive results on the unaligned images, as shown in Figure 7b,c. At the same time, dim target and noise cause miss detection, which is the main reason for a low recall rate. Figure 8 displays some cases of dim supernovae, and the target is marked by a red arrow. The above results show that the model has defects in the detection of unaligned and dim targets. Therefore we need to pay more attention to alignment and exposure in the data pre-processing stage of the supernovae detection system.

## 7. Conclusions

In this paper, we studied the problem of supernovae detection, and compare two potential object detection frameworks on this task. We built two new supernovae detection datasets, which consist of 12,447 images and 716 images respectively. We believe such datasets will benefit the relevant research community. The FCOS achieves the performance of 99.6% precision and 98.6% recall on the PS1-SN dataset and 84.9% precision and 51.4% recall on the PSP dataset. Experimental results show that the FCOS performs better than YOLOv3 on both datasets, so the FCOS selected as the baseline is used to further improve the performance for supernovae detection according to the feature of the supernova. In our experiment, the original PSP dataset is augmented to 12,384 images with a series of techniques and generates obvious improvement on our task. Here we have tested different attention blocks and studied the effect of different input image sizes on the detecting performance. Eventually, the FCOS with spatial attention module and bigger size of input reaches 96.2% precision and 64.3% recall, which is the best performance we can get in our work. Besides, we have tried some other ideas, but they do not have the effect of improving performance. By using the object detection framework, we can implement the automatic processing of supernovae detection tasks and thus accelerate the processing pipeline of observation data.

Object detection framework is a flexible object extractor and classifier. Especially in the complex scene, the automatic extraction and detection of objects are particularly important. Compared with the traditional neural network with only a few layers, the object detection framework can achieve high-precision and real-time detection based on deep convolutional networks to deal with the information represented by complex circumstances. We suggest that future research should be based on a deeper convolution network and adopt small target detection technology. One limitation of our research is that we do not have access to multi-channel images for these targets. Multi-channel images such as UVBIR images are often used in astronomical images. These images contain infrared and ultraviolet channels that may provide more information about supernovae. Therefore, in our future research, we will seek opportunities to obtain multi-channel images from real scenes to enhance our datasets. Besides, we found image size has an impact on the detection performance of FCOS. We will investigate how to automatically and efficiently select the best image size in the future.

## Figures and Tables

**Table 1:** The results of the different methods on the two datasets.

Framework	Dataset	Precision	Recall	F1
DH [12]	PS1-SN	0.981	0.984	0.982
CAP [14]	PS1-SN	0.996	0.980	0.987
YOLOv3 [27]	PS1-SN	0.994	0.988	0.991
FCOS [10]	PS1-SN	0.996	0.986	0.994
YOLOv3	PSP-Ori	0.618	0.764	0.683
FCOS	PSP-Ori	0.879	0.579	0.713

**Table 2:** Training parameters.

Parameter	YOLOv3	FCOS
learning rate	$1\times 10^{-2}$	$1\times 10^{-4}$
momentum	$9\times 10^{-1}$	-
decay	$1\times 10^{-4}$	$1\times 10^{-4}$
batch size	16	16
epoch	30	16
image size	416	416

**Table 3:** Performance of the FCOS with different improvement techniques on the PSP dataset.

Augmented	Max Size	Channel Attention	Spatial Attention	CBAM	Precision	Recall	F1
-	416	-	-	-	0.866	0.536	0.662
✓	416	-	-	-	0.923	0.638	0.754
✓	416	✓	-	-	0.912	0.623	0.740
✓	416	-	✓	-	0.904	0.609	0.728
✓	416	-	-	✓	0.879	0.579	0.698
✓	800	-	-	-	0.926	0.645	0.760
✓	960	-	-	-	0.957	0.642	0.768
✓	960	✓	-	-	0.962	0.643	0.771
✓	960	-	✓	-	0.960	0.645	0.772
✓	960	-	-	✓	0.954	0.608	0.743

**Table 4:** Performance of the YOLOv3 with different improvement techniques on the PSP dataset.

Augmented	Input Size	Channel Attention	Spatial Attention	CBAM	Precision	Recall	F1
-	416	-	-	-	0.618	0.764	0.683
✓	416	-	-	-	0.554	0.800	0.654
✓	416	✓	-	-	0.585	0.820	0.683
✓	416	-	✓	-	0.569	0.774	0.656
✓	416	-	-	✓	0.480	0.636	0.547
✓	800	-	-	-	0.780	0.759	0.769
✓	960	-	-	-	0.742	0.794	0.767

**Table 5:** Performance of FCOS on the hybrid datasets with different blending ratios.

Test Set	Training Set		Precision	Recall	Cls F-Score
	PS1-SN	PSP-Aug			
PS1-SN	100%	-	0.996	0.995	0.994
PS1-SN	100%	5%	0.997	0.984	0.994
PS1-SN	100%	100%	0.997	0.981	0.995
PSP-Aug	-	100%	0.866	0.536	0.837
PSP-Aug	10%	100%	0.840	0.511	0.828
PSP-Aug	100%	5%	0.704	0.389	0.748
PSP-Aug	100%	100%	0.800	0.472	0.786

## Data Availability

The datasets in this paper can be obtained from the following link: https://github.com/KopiSoftware/Supernova-Detection-Datasets, accessed on 16 November 2020.
